# Twelve tips for structuring classes in higher education: lessons learned from a biology premedical class

**DOI:** 10.15694/mep.2019.000129.1

**Published:** 2019-06-11

**Authors:** Archita Chandra, Genevieve Schmitt, Rohini Ganjoo

**Affiliations:** 1 George Washington University

**Keywords:** Medical education, active learning, flipped classroom, post baccalaureate premedical biology, undergraduate education, biology, student engagement, in-class activities, pedagogy, teaching

## Abstract

This article was migrated. The article was marked as recommended.

The efficacy of flipped classrooms on student learning is dependent on student motivation. We conducted a study on the impact of the flipped classroom on student performance and satisfaction among highly motivated students enrolled in a post-baccalaureate pre-medicine program for career-changers. Surprisingly, students in both traditional and flipped classrooms indicated lecture was the largest contributor to their learning. Additionally, students in the flipped classroom highlighted specific activities most useful to their engagement, comprehension, and exam performance. Thus for heterogeneous student populations in higher education, didactic lecture remains an integral component of learning. Based on our study and relevant literature, we suggest tips on how to effectively structure courses in higher education that are tailored to the learning needs and motivations of your specific student demographic.

## Introduction

More students than ever before are attending college with a projected 17.4 million students enrolled in undergraduate institutions by 2027 ( National Center for Education Statistics, 2018). The increasing number of students has been marked by advances in our understanding of pedagogy and added pressure on educators to reconfigure curriculum and teaching styles to benefit today’s student body. Traditionally, the didactic teaching style was an effective method to impart knowledge. However, an exponential growth of instructional strategies, including cooperative learning, visualization, differentiation, and implementation of technology, have improved learning opportunities ( Dunlosky *et al*., 2013). The cumulative stress of juggling educational requirements, and the sheer number of teaching strategies available, can be overwhelming, leading to significant teacher burnout and exhaustion ( Taris, Schreurs and van Iersel-van Silfhout, 2010). Flipped classroom methods typically include readings, videos and instructions for students to review before attending class, while time spent in the classroom consists of activities meant to clarify and cement the knowledge in a collaborative environment ( Tucker, 2012). There are mixed findings about the effectiveness of the flipped model. Studies show them to be effective for student knowledge ( O’Flaherty and Phillips, 2015); however, studies on baccalaureate nursing students found no differences in overall performance by flipping the classroom, implying that traditional didactic lectures were considered equally effective for student learning (Geist et al., 2015; Harrington *et al*., 2015). Interestingly, Missildine et al. (2013) noted nursing students actually preferred lecture-based learning, as they felt more confident in learning from a subject matter expert. These studies indicate that the efficiency of an educational technique varies widely depending on the student population.

To explore this further, we conducted a study of two student cohorts enrolled in a biology course that is part of a post-baccalaureate pre-medicine program (PBPM) program. These students did not have the prerequisite courses in the life sciences to take the Medical College Admission Test (MCAT). One cohort was taught using a traditional didactic method, with a majority of the concepts taught through lecture. The classroom was flipped for the second cohort by conducting 50% less lecture time and devoting in-class time to various active learning techniques including educational games, like jeopardy and pictionary, case study discussions, questionnaire worksheets, concept maps, student presentations, connection assignments and group work. No significant differences in performance were observed between the two cohorts as measured by periodic and final exams. Students reported on surveys administered before the end of the course that they most preferred in class lectures. This is consistent with reports that students similar to the PBPM students with high extrinsic motivation, advanced degree levels, and science-focused curriculum, do not necessarily benefit by the flipped classroom strategy ( Abeyskera and Dawson, 2015; Taylor, *et al*., 2014). Thus, it may be more effective to implement aspects of the educational strategies rather than use a single method (DeLozier and Rhodes, 2016). We describe how the flipped style can be adapted to student needs and hope that the tips will lower stress by removing perceived barriers educators face in creating a successful curriculum.

## Twelve Tips

### Tip 1. Know your subject matter

Faculty are subject matter experts, well versed in their field and eager to share their knowledge with students. However, narrowing the vast knowledge of a subject to the specific needs of the course is important. For example, the PBPM program provides prerequisite science courses for students who want to pursue medicine. Program faculty filter content so students do not spend time on material that may be irrelevant to their needs. Plant biology is fascinating, but not important for student performance on the MCAT or medical school. A fun and effective way to incorporate plant content may be through an assignment where they have to research medicinal herbs or the validity of plant use in medicine. This exposes students to the topic without bogging them down with extra details. If you are unclear on how to distill content, ask yourself ‘Will this be important for the student in 3 -5 years?’ to help tailor the course.

### Tip 2. Know your general class demographic

Appreciating the diversity and demographic backgrounds of your students helps to gauge their needs and expectations of the course and to create relevant and engaging teaching material. In many non-degree courses, masters, doctoral, and professional programs, students are returning to higher education after years in the professional world, bringing valuable work experience and a global perspective. This multifaceted outlook is relevant for both liberal arts and science courses. PBPM instructors often incorporated discussions on access to healthcare stratified by multiple factors, such as race, ethnicity, and culture. These discussions allow students to share their diverse perspectives on how topics relevant to the course affects different groups.

### Tip 3: Estimate student motivation

Predicting the educational motivation of students helps in preparing activities or lectures effectively. Extrinsically motivated students learn for rewards, like grades, and complete activities for gains or benefits in their performance, while intrinsically motivated students learn the material for their own interest, so their participation in activities is not influenced by rewards ( Taylor, *et al*., 2014). Students in primary or secondary education show more intrinsic motivation, whereas students in higher education show more extrinsic motivation ( Abeyskera and Dawson, 2015). Estimating educational motivation helps to determine class structure. Incorporating the flipped classroom can be more helpful for students with more intrinsic motivation, whereas students with more extrinsic motivation prefer traditional didactic lectures for their learning ( Zainuddin and Perera, 2017). By understanding that students in our biology course in the PBPM consisted mainly of career changers who needed to complete prerequisites for medical school, we expected our students to have more extrinsic motivation during the semester, which aided in course structure and design.

### Tip 4. Know your resources → don’t reinvent the wheel, redesign it

A ‘medical education active learning’ search in Google Scholar reveals more than 3,100,000 results. Plenty of resources, classroom activities and curricula are readily available to create engaging curricula that maximize student learning. Organizations compile resources and blogs dedicated to education, metacognition, and medical teaching. For example, clinical case studies for discussion in the biology courses of the PBPM were often obtained from the National Center for Case Study Teaching in Science, which aligned with the needs of the pre-medical students and goals of the program. Do not feel the need to create an assignment that is mind blowing in what it can achieve; use easily available resources instead.

### Tip 5. Find practical applications of your material and resources

Focus on identifying activities that students find practical in their lives. Studies have shown that student engagement increases with more relatable content ( Aish, Asare and Miskioglu, 2018; Stiles, 2013). Creating personal connections to the content and exposing students to current topics engages them. For the PBPM biology course, the frequent, clinical applications of course content were highlighted in assignments. As one student noted “The connection assignment was great for pre-med students to understand the ‘so what’ and especially helpful for those who did not come from medical backgrounds.” Protein assay techniques were explained using data from diabetes studies, a common disease with which some students had personal experience. Incorporating current drug research and ethical dilemmas were important in informing students of new developments, while relaying complex topics in relevant, practical, and engaging ways.

### Tip 6. Use technology to your advantage

Technology pervades almost every line of work, and exposure to it can be useful in the long term ( Bybee and Fuchs, 2006); thus, it makes sense to take advantage of this resource. Allowing students to upload and submit pictures of written assignments through the university’s learning management system can reduce confusion; it is proof that the student submitted assignments on time, ensures both parties have a copy, and allows grades to be easily displayed. Lecture recording technology provides flexibility for students to access lectures outside the classroom. Interestingly, Gupta and Saks (2013) showed that a majority of first year medical students used recorded lectures as supplements to physical lectures, whereas a majority of second year medical students used recorded lectures as replacements for physical lectures. How recorded lectures were used depended primarily on “the quality of the lecturer” (p. 767) and the content of material; i.e., recorded lectures were more likely to be used as a supplement to lecture for content that had more visual elements, such as anatomy. Thus, using technology to aid student learning and preferences, while effectively relaying content, is ideal.

### Tip 7. Be flexible in choosing what resources to incorporate in your plans

While combing through the multitude of resources available, it is easy to get intimidated and overwhelmed by what and how many resources to incorporate. However, do not feel the need to incorporate every resource or teaching aid. Allocating class time between lecture and active learning exercises is as important as knowing the subject matter ( Ballen, *et al*, 2017). Assignments should be tailored to benefit different student learning styles. Our students self-reported that questionnaire worksheets and learning games were most engaging and most useful for exam preparation.

### Tip 8. Plan for contingencies

Life happens! Extreme weather cancels class, you fall sick, or the printer breaks down right before class. Whatever the reason, make your syllabus adaptable to unforeseen events. Plan for these contingencies by forming a flexible, alternate schedule. What lectures and topics could be grouped together in a combined lecture for a make-up class? Virtual lectures simulating in-class instruction allow students to interact with the instructor and ask questions in real time. What concepts can students learn in online, at-home lectures or in a self-study manner to make up for lost class time? Being adaptable to possible adverse events will allow you to provide class content to students regardless of circumstances.

### Tip 9. Get to know the reality of your class

Student learning builds on prior knowledge. To illustrate this, consider administering a pre-test of course material, with 5-10 open response questions. For larger classes, use Poll Everywhere/Kahoot/Clicker Questions to easily gauge the foundational knowledge of students. “A 5 question quiz on the two chapters we read for the day of lecture kept us on our toes,” remarked a student. Short quizzes administered at the beginning of class can gauge students’ prior understanding of concepts and help students identify gaps in content comprehension. Such activities help students better understand your teaching style and expectations for them during the course. Being cognizant of students’ prior knowledge helps better tailor teaching strategies and explanations of the material to enhance content comprehension.

### Tip 10. Be transparent with students about your support for their learning

Being transparent with students builds trust and a supportive environment. Take students into confidence at the start of your course and share how you plan to support their learning. This can be accomplished by reviewing the course schedule and highlighting important activities or discussions, or by providing a cursory overview of how much class time will be devoted to didactic lecture versus active learning exercises. Regardless, being transparent shows your sincerity for students’ success and your respect for them as responsible, adult learners. This collaborative environment facilitates the understanding of your expectations of students throughout the course, leaving little room for misinformation or confusion.

### Tip 11. Check in to assess student satisfaction

Monitoring student grades on exams and assessments is typically conducted throughout a course. Gauging student satisfaction and perceived learning, however, may be more challenging to conduct yet equally important. Have intentional, periodic check-ins with students via short discussions, 5-minute exercises where they anonymously write two things they like and dislike about class, or by more detailed surveys on students’ self-reported satisfaction, learning, and comprehension. Rather than relying only on end of course evaluations, satisfaction assessments should be done at least once in the middle of the course to allow concurrent modifications to the class, if necessary. For example, in-class student presentations on content was theoretically a great idea implemented in our biology class to ensure students reviewed content before class. However, informal feedback from students after class revealed that these presentations were not valuable, resulting in a replacement of the presentations with other activities. We also learned that a majority of students preferred more classroom time to be spent on lecture, as they valued class discussions generated during the lecture that helped clarify concepts otherwise missed during class activities. When asked how they would structure class, one student noted, “I would make class 75% lecture and 25% activities to enhance the learning experience and engagement,” while another said, “I would weigh heavily on lecture, mainly because that is my best method of learning.” This feedback helped tailor the class and showed students that their suggestions were heard and incorporated, which further boosted motivation and satisfaction.

### Tip 12. Review your strategies and adjustments for future renditions

Finally, take time to create a supportive learning community for students by recognizing the reasons behind their preferences and ideas. Students engage with the content and their peers to create a collegial class atmosphere when they perceive faculty as an extension of their team. Give serious consideration to end-of-semester student feedback and appreciate student compliments. Reflecting on strategies and experiences is valuable in improving communication and teaching methods for future renditions of the course.

## Summary

Undergraduate premedical education is vibrant and unique; with a diverse student population, it can be challenging for faculty to navigate. We hope these tips will guide educators who have not yet included active learning in their classes, those who have tried it without positive results, and those who would like to tweak their teaching approach. The tips are important to tie seemingly disjointed pieces of a classroom into a nurturing environment that helps students reach their potential and goals, while satisfying the faculty mission of contributing to a well prepared future medical student population that is grounded with knowledge and familiar with various pedagogical styles.

## Take Home Messages

To build an effective engaging course for students in higher education:

• Know how to make your content applicable for your student demographic.
• Predict your students’ educational motivation to help design your course, adapt available resources, and use technology smartly.
• Be open to incorporating student feedback to your course structure during the semester, and be adaptable to unforeseen events.
• To boost motivation in class, get to know your students, their learning styles, and their satisfaction throughout the course.
• At the end, reflect on all the pros and cons of your journey to have smooth sailing in the future!

## Notes On Contributors

***Rohini Ganjoo***, PhD, is an Assistant Professor in the George Washington University. As the founding faculty she developed the curriculum and content for Biology for the PBPM students and can be contacted for more information on the activities used in this study. ORCID number: 0000-0001-7185-7747

***Archita Chandra***, B.S., was a student and Teaching Assistant at the George Washington University. She performed data collection and analysis, and presented results at the GW Research Days.

***Genevieve Schmitt***, B.S., was a student and Teaching Assistant at the George Washington University, and worked to create new content and implement more technology to aid student learning and teacher efficiency.
