# Supplementary material for: Twelve tips for structuring classes in higher education: lessons learned from a biology premedical class
Source: MedEdPublish (2016). 2019 Jun 11;8:129. [Version 1] doi: 10.15694/mep.2019.000129.1 (PMC10712471; doi:10.15694/mep.2019.000129.1)
Supplement: Peer review history

# Reviewer report: Reviewer response for version 1

**DOI:** 10.21956/mep.19864.r30010

**Published:** 2019-08-08

**Authors:** Irine Sakhelashvili

**Affiliations:** 1 European University

This review has been migrated. The reviewer awarded 5 stars out of 5 I found this article very useful and informative. It is always difficult to find a balance between didactic and modern methods while teaching, especially in high medical education. As it was mentioned by the authors it was surprising the findings that students indicated the didactic lecturers as a largest contributor to the learning. I would like to know the content of the questionnaire as whole design and results of survey for better understanding the results. As for the tips they are most valued part of the presented paper. They should be considered regardless the subject conducted. Thank you for sharing an experience.

Reviewer Expertise:

NA

No decision status is available

# Reviewer report: Reviewer response for version 1

**DOI:** 10.21956/mep.19864.r30006

**Published:** 2019-07-30

**Authors:** Barbara Jennings

**Affiliations:** 1 Norwich Medical School

This review has been migrated. The reviewer awarded 2 stars out of 5 The authors of this article share their experiences and conclusions from working in undergraduate premedical education, which they describe as a vibrant and unique environment. They explore the use of flipped classrooms and make 12 suggestions about how to create a successful curriculum. The authors remind us of some core principles for the medical educationalist, in their useful list of tips: such as the importance of shaping the curriculum, so that the outcomes are in keeping with the future (knowledge and skills) needs of the learners. Also, the successful didactic teacher will need to understand the prior knowledge of their cohorts, and how to encourage their learning effectively.I noted some particular problems when I read the introduction that could be addressed in a revised version of the article. The authors were interested in the learning gain and acceptability of flipped lectures but in their discussion of the background literature they did not fully define the flipped lecture. Furthermore there were some emphatic statements with no supporting evidence presented; e.g. “didactic teaching style was an effective method to impart knowledge….” The educational literature about the use of flipped classrooms in clinical education was also not presented systematically. There is a study conducted by the authors that is outlined in the introduction, but with insufficient detail for the reader to fully understand it. Did the learners in the second cohort receive online didactic teaching material or pre-reading (both of which is usual for flipped classrooms) or was it just the amount of activity in the face to face session that was varied? Without this detail or the full results it is hard for the reader to consider your interpretation of the case study.Thank you for sharing your experiences and I enjoyed reading your article; a revised article could address the gaps discussed above.

Reviewer Expertise:

NA

No decision status is available

# Reviewer report: Reviewer response for version 1

**DOI:** 10.21956/mep.19864.r30008

**Published:** 2019-06-14

**Authors:** John Gillum

**Affiliations:** 1 GWU

This review has been migrated. The reviewer awarded 5 stars out of 5 Very informative! Obviously, we need to see more data to outcomes for method of pedagogy. Certainly the author gives some insightful tips. Future educational researcher will find this helpful.

Reviewer Expertise:

NA

No decision status is available

# Reviewer report: Reviewer response for version 1

**DOI:** 10.21956/mep.19864.r30007

**Published:** 2019-06-11

**Authors:** Teresa Donze-Reiner

**Affiliations:** 1 West Chester University

This review has been migrated. The reviewer awarded 4 stars out of 5 Please provide data, graphs, and/or figures to document the study you conducted on the two cohorts. Also, how many students were in each cohort? What statistical test was used to prove/disprove significance in learning strategies?On Tip 10, I do not understand the statement "Take students into confidence at the start of your course...."Overall, good content and review of tips that are beneficial for teaching Post-bac students.

Reviewer Expertise:

NA

No decision status is available

# Reviewer report: Reviewer response for version 1

**DOI:** 10.21956/mep.19864.r30009

**Published:** 2019-06-11

**Authors:** Felix Silwimba

**Affiliations:** 1 University of Lusaka

This review has been migrated. The reviewer awarded 5 stars out of 5 informative and useful tips for lecturing approaches.

Reviewer Expertise:

NA

No decision status is available
